# Metasurface enabled quantum edge detection

**DOI:** 10.1126/sciadv.abc4385

**Published:** 2020-12-16

**Authors:** Junxiao Zhou, Shikai Liu, Haoliang Qian, Yinhai Li, Hailu Luo, Shuangchun Wen, Zhiyuan Zhou, Guangcan Guo, Baosen Shi, Zhaowei Liu

**Affiliations:** 1 Key Laboratory of Quantum Information, University of Science and Technology of China, Hefei, Anhui 230026, China and Synergetic Innovation Center of Quantum Information and Quantum Physics, University of Science and Technology of China, Hefei, Anhui 230026, China.; 2 Key Laboratory for Micro-/Nano-Optoelectronic Devices of Ministry of Education, School of Physics and Electronics, Hunan University, Changsha 410082, China.; 3 Department of Electrical and Computer Engineering, University of California, San Diego, 9500 Gilman Drive, La Jolla, CA 92093, USA.; 4 Interdisciplinary Center for Quantum Information, State Key Laboratory of Modern Optical Instrumentation, ZJU-Hangzhou Global Science and Technology Innovation Center, Zhejiang University, Hangzhou 310027, China.

## Abstract

Metasurfaces consisting of engineered dielectric or metallic structures provide unique solutions to realize exotic phenomena including negative refraction, achromatic focusing, electromagnetic cloaking, and so on. The intersection of metasurface and quantum optics may lead to new opportunities but is much less explored. Here, we propose and experimentally demonstrate that a polarization-entangled photon source can be used to switch ON or OFF the optical edge detection mode in an imaging system based on a high-efficiency dielectric metasurface. This experiment enriches both fields of metasurface and quantum optics, representing a promising direction toward quantum edge detection and image processing with remarkable signal-to-noise ratio.

## Teaser

Remotely switchable edge imaging is demonstrated on the basis of the metasurface illuminated by polarization-entangled photons.

## INTRODUCTION

Photonic metasurfaces, two-dimensional ultrathin arrays of engineered metallic or dielectric structures, are versatile optical components enabling electromagnetic field manipulation of the local phase, amplitude, and polarization (*1*–*4*). These capabilities are typically developed for various applications in the regime of classical optics. Quantum entanglement is an essential source of quantum optics for many applications, such as quantum cryptography (*5*, *6*), teleportation (*7*–*9*), superresolving metrology (*10*), and quantum imaging (*11*). In particular, in the field of quantum imaging, spatial intensity correlations between photon pairs can be exploited to surpass the classical limits of imaging (*12*–*14*). Besides, the introduction of quantum image processing techniques illuminated with heralded single photons reveals the superior antinoise capacity for photon-limited imaging (*15*). Recent efforts indicate a trend to combine the metasurface with entangled photons for various potential applications in quantum optics (*16*–*20*).

In another context, edge detection is one of the most common operations in image processing, which attempts to define the boundaries between regions in an image. It is a basic tool in the field of machine and computer vision (*21*), a preprocessing step for automated characterization in medical images operation (*22*, *23*), and a critical component of autonomous vehicles (*24*, *25*). Compared with traditional digital methods, the analog technique has high-speed and power-saving advantages. Therefore, various analog edge detection approaches have been proposed (*26*–*35*), including by using metamaterials and metasurfaces (*36*–*39*). However, edge detection based on compact metasurface has never been demonstrated in the field of quantum optics. Since quantum entanglement owns indistinguishable information before measurement and instantaneous action at a distance, one can expect that the metasurface-enabled edge detection used in quantum optics will offer possibilities for remotely controllable image processing and cryptography.

In this work, we formulate and realize polarization-entangled photon source and high-efficiency metasurface enabled switchable optical edge detection. By selecting a proper polarization state in the heralding arm of the entangled photon source, either normal image or edge image is obtained. It can be regarded as an entanglement-assisted remote switch for edge detection. Compared to the case by using classical light sources, the quantum edge detection scheme shows a high signal-to-noise ratio (SNR) at the same photon flux level.

**Fig. 1:**
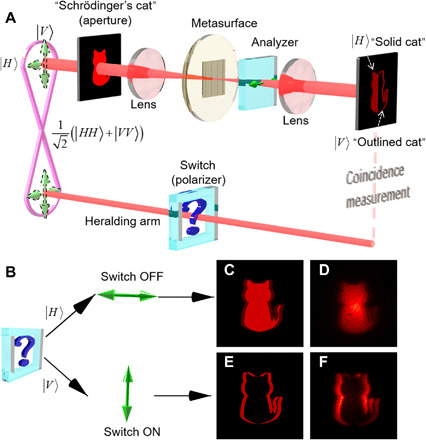
The schematics of a metasurface enabled quantum edge detection. (**A**) The metasurface is designed to perform edge detection for a preferred linear polarization. |*V*〉, i.e., polarization state is orthogonal to the analyzer. The dashed light red line stands for the electrical path. The question mark means that polarization selection of idler photons of the heralding arm is unknown. If the Schrödinger’s cat is illuminated by unknown linear polarization photons from the polarization entangled source, the image would be a superposition of a regular “solid cat” and an edge-enhanced “outlined cat.” (**B**) The switch state ON or OFF of the heralding arm. When the idler photons of the heralding arm are projected to |*H*〉, it indicates the switch OFF state and leads to a solid cat captured. While the heralded photons are projected to |*V*〉, an edge-enhanced outlined cat is obtained with the switch ON state. (**C** and **D**) The calculated and experimental results of a solid cat, respectively. (**E** and **F**) The calculated and experimental results of the edge-enhanced outlined cat, respectively.

We use the “Schrödinger’s cat” (refer to fig. S1) to illustrate the expected performance of the switchable quantum edge detection scheme. Here, let us first briefly review the basic principle of edge detection based on the classical continuous-wave (CW) light illumination (*36*). As schematically shown in the top portion of Fig. 1A, the edge detection imaging arm is independent of the entangled source, heralding arm, and coincidence measurement components. When the incident photons have a horizontal polarization state, the beam of illuminated cat goes through an engineered metasurface and then separated into a left- and a right-handed circular polarized (LCP and RCP) image with a predesigned horizontal shift. The overlapped LCP and RCP components will pass through the analyzer orientated along the same horizontal direction, resulting in a complete “solid cat.” If the incident photons are vertically polarized, the overlapped LCP and RCP components will be recombined to a linear polarized (LP) component and completely blocked by the analyzer, which only leaves out the edges ending up with an “outlined cat” (*36*). In this work, polarization-entangled photons with the state of $\frac{1}{\sqrt{2}}(|\mathrm{HH}\rangle +|\mathrm{VV}\rangle )$ are used as an illumination source to implement the quantum switchable edge detection. The heralding arm is introduced and serves as an external trigger to acquire coincidence images between pairs of entangled photons. When the incident and heralded photons (which will be explained later) are entangled and without knowing their polarization states, the image should be a quantum superposition of a “solid cat” and an “outlined cat.” If the polarization state of the incident photon is selected and triggered by a heralding arm, the image can be switched between a regular mode of a solid cat and an edge detection mode of an outlined cat (refer to note S1 and fig. S2).

## RESULTS

### Experimental setup

**Fig. 2:**
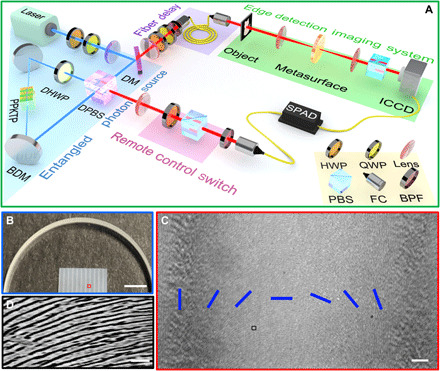
Experimental setup and sample characterization. (**A**) Experimental setup of metasurface enabled quantum edge detection. BDM, broadband dielectric mirror; PBS, polarization beam splitter; DM, dichromatic mirror; FC, fiber coupler; BPF, band-pass filter; ICCD, intensified charge coupled device. By pumping a nonlinear crystal (type II phase-matched bulk PPKTP crystal) with a 405-nm laser, pairs of orthogonally polarized photons with 810-nm wavelength are generated through the spontaneously parametric down-conversion process. The blue (red) light path presents the 405-nm (810 nm) light. Edge detection switch is on the heralding arm. An edge detection imaging system is on the imaging arm. (**B**) Photograph of the partial metasurface sample. Scale bar, 4 mm. (**C**) Polariscopic analysis characterized by crossed linear polarizers of the sample area marked in 2a. The blue bars indicate the orientation of rotated nanostructures in one period, which represents the Pancharatnam-Berry phase induced by the laser writing dielectric metasurface. Scale bar, 50 μm. (**D**) The scanning electron microscopy image of the sample area marked in (C). Scale bar, 1 μm. Photo credit: Junxiao Zhou, University of California, San Diego.

A schematic diagram of the experimental setup is sketched in Fig. 2A. Polarization-entangled photons are generated via spontaneous parametric down-conversion process in a 20-mm-long type II phase-matched periodically poled KTiOPO_4_ (PPKTP) crystal embedded in a Sagnac interferometer. The temperature of the PPKTP crystal is set at 17°C, the photon degenerate wavelength temperature, by a homemade temperature controller with a stability of 0.002°C. Two broadband dielectric mirrors and a dual-wavelength (405 and 810 nm) polarization beam splitter (DPBS) form the self-stable Sagnac interferometer. The pump beam is from a CW single-frequency diode laser at 405 nm (Toptica, TOP mode-405-HP_40116) and focused by a pair of lenses with optimized focal lengths to get a beam waist of approximately 40 μm at the center of the crystal. The combination of a quarter-wave plate (QWP) and a half-wave plate (HWP) in front of the Sagnac loop is used to balance the pump power between clockwise and counterclockwise directions. A dual-wavelength HWP placed in the Sagnac loop is fixed at 45° to obtain the horizontal polarization of the counterclockwise pump directions in front of the crystal. The down-converted photon pairs pumped by two counter-propagating beams are separated by DPBS. They are collimated by two lenses, of which one goes to a fiber coupler into the imaging arm, and the other goes to the heralding arms, respectively.

The combination of one HWP and one PBS in the heralding arm is used to select horizontal or vertical polarization of photons for switching between the normal imaging and the edge detection mode. After that, the heralded photons are detected by a single-photon avalanche detector (SPAD) with a detection efficiency of 60% at 810 nm, which acts as a trigger for the intensified charge-coupled device camera (ICCD). Photons in the imaging arm with the mixed polarization state of ∣*H*〉 or ∣*V*〉 are injected into a 4f imaging system. Note that the metasurface is placed on the Fourier plane in the imaging arm, which modifies the Fourier space spectrum of the object. The distribution of the object, the first lens, the metasurface, the second lens, and ICCD are equidistant with a distance of 100 mm (the focal length). An 810-nm PBS as an analyzer placed in front of the ICCD implements the horizontal LP measurement of all the passing photons.

It should be noted that to make sure that the photons detected by the heralding detector and the ICCD camera are from the same photon pair, the photon of the imaging arm has to be delayed with an additional optical path length to match the electrical delayed response of the heralding trigger signal. By calculation, a 15-m single-mode fiber as the optical delay is added to the imaging arm. It ensures that for each photon detected by ICCD camera, its sibling photon is recorded by the SPAD at the same time. Also, to solve the polarization dephasing caused by fiber transmission, the target Bell state is correlated back by another combination of HWP and QWP in front of the object. Here, to eliminate undesired fundament light, two band-pass filters of 810-nm wavelength with a 40-nm bandwidth are set in front of both heralding and imaging arms, respectively.

Figure 2 (B to D) shows the characterization of the metasurface sample. The metasurface was designed by using the Pancharatnam-Berry phase (*40*, *41*) and was fabricated by scanning a femtosecond pulse laser inside a silica slab (around 50 μm beneath the surface). The self-assembled nanostructures inside of the silica slab are formed under intense laser irradiation. By varying the laser polarization gradually, the orientation of nanostructures will be gradually varied. For our metasurface, the corresponding transmission efficiency (the ratio between the transmitted power and the incident power) is measured at the working wavelength of 810 nm as high as 90%. The measured conversion efficiency (the ratio of RCP and LCP components to the incident LP beam) is close to unity at the working wavelength. More sample fabrication details could be found in our previous work (*42*, *43*). Polariscopic analysis characterized by crossed linear polarizers on the sample area marked in Fig. 2B is shown in Fig. 2C. The fingerprint-like features could be regarded as nanostructures with different orientations providing the needed geometric phase, as can be clearly seen from a scanning electron microscopy image in Fig. 2D.

### The property of polarization-entangled photon pairs

First, a brief introduction of the quantum state preparation is provided. The polarization-entangled degenerate photon pairs are generated from the Sagnac loop (*44*, *45*). The output state of the Sagnac loop could be written as $|\Psi \rangle =\frac{1}{\sqrt{2}}(|\text{HV}\rangle +e^{i\theta }|\text{VH}\rangle )$ (*46*). The achieved value of the relative phase θ is 0 or π, corresponding to the Bell states ∣Ψ^+^〉 or ∣Ψ^−^〉, respectively. Here, the Bell state $|\Phi^{+}\rangle =\frac{1}{\sqrt{2}}(|\mathrm{HH}\rangle +|\mathrm{VV}\rangle )$ is used for this work by adjusting the combination of HWP and QWP in front of the imaging arm. To characterize the quality of the generated polarization-entangled state $|\Phi^{+}\rangle =\frac{1}{\sqrt{2}}(|\mathrm{HH}\rangle +|\mathrm{VV}\rangle )$, two-photon polarization interference and quantum state tomography are performed.

**Fig. 3:**
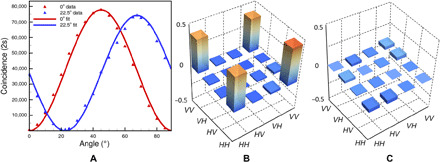
Characterizations of the entangled source. (**A**) Coincidence counts as a function of the HWP angle θ_2_ at one output port in 2 s. The red (blue) color of count data and interference corresponds to horizontal (diagonal) projection bases. The solid lines are sinusoidal fits to the data, error bars are estimated by assuming Poisson photon statistics in photon counting. Error bars are obtained from multiple measurements. (**B** and **C**) The real and imaginary parts of the reconstructed density matrix ρ of the two-photon states, respectively.

The image quality of quantum-switchable edge detection depends on the prepared quantum state. Hence, the characterization of the entangled source is indispensable. For measuring the polarization interference fringes, the HWP rotation angle θ_1_ of the imaging arm is fixed at 0 or 22.5°, and then two-photon coincidence will be a function of the HWP rotation angle of the heralding arm, generally given by *C*(θ_1_, θ_2_) = ∣⟨θ_1_ ∣ ⟨θ_2_ ∣ Φ^+^⟩∣^2^ ∝ sin^2^[2(θ_1_ − θ_2_)] under the current experimental condition. The sinusoidal quantum interferences, shown in Fig. 3A, reveal the polarization correlations between photon pairs measured in the horizontal (θ_1_ = 0°) and diagonal (θ_1_ = 22.5°) polarization basis. Note that for measuring the two-photon coincidence, we replace the edge detection imaging system by a SPAD. The interference visibility is calculated as $V=\frac{C_{\text{max}}-C_{\text{min}}}{C_{\text{max}}+C_{\text{min}}}$, in which the *C*_max_ and *C*_min_ are maximum and minimum coincidence counts, respectively. At last, the raw interference fringe visibilities are calculated as 96.7 ± 0.1% in the +45°/−45° basis, and 96.4 ± 0.1% in the *H*/*V* basis, respectively, which both exceeded 71%, the bound required to violate the Bell’s inequality (*47*).

Then, the entanglement quality of the two-photon state is quantified by quantum tomography and the reconstructed two-photon density matrix measurements (*48*). The fidelity of the reconstructed density matrix of our present source is defined as *F* = 〈Φ^+^∣ρ_exp_∣Φ^+^〉, where ∣Φ^+^〉 is the ideal Bell state. As shown in Fig. 3 (B and C), the fidelity of the reconstructed density matrix for our present source is found to be 93.5 ± 0.013% according to the reconstructed density matrices. The characterizations of polarization entangled source are performed with a pump power of 7 mW, the single count rate in the heralding/imaging arm is 480/240 kHz, and the heralding efficiency is about 11.7%.

### The entanglement enabled quantum edge detection

**Fig. 4:**
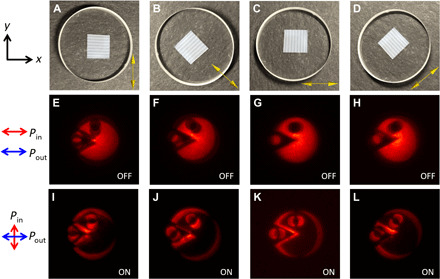
The switchable edge detection demonstration. (**A** to **D**) The metasurface sample orientation, which is aligned with the *xy* plane. The inset yellow arrows indicate the phase gradient direction of the metasurface. (**E** to **H**) The images of the whole object comprising the separated LCP and RCP components, which is the OFF state of the edge detection mode. (**I** to **L**) The images reveal edges along different directions, which is the ON state of the edge detection mode. Photo credit: Junxiao Zhou, University of California, San Diego.

After having confirmed the quality of generated polarization-entangled photon pairs, switchable quantum edge detection is demonstrated. Here, the heralding arm serves as an external trigger, of which photons are mixed with unknown polarization states. They can be prepared in horizontal or vertical linear polarization states by polarization postselection using an HWP and a PBS of the heralding arm. When the polarization state of photons in the heralding arm is measured along the horizontal direction, the correlated polarization of the illumination photons in the imaging arm will also be ∣*H*〉. These photons are coupled into fiber and sent to the edge detection image system. The final alternative image between the edge detection mode and the regular mode is captured by the heralding-triggered ICCD. As shown in Fig. 4 (E to H), two overlapped images with a tiny shift are acquired, of which the shift direction is aligned with the phase gradient direction of the metasurface [the yellow arrows in Fig. 4 (A to D)]. It is worth noting that the shift of two overlapped images could be decreased by increasing the period of the metasurface structure, so that high-resolution edge detection can be achieved, as demonstrated in our previous work (*36*). Since the illumination photons are along the same orientation with the analyzer, both images are recorded with dimmer edges along the selected directions. After postselecting trigger photons in ∣*V*〉 polarization in the heralding arm, the image photons will be captured only in ∣*V*〉 polarization, which is orthogonal to the orientation of the analyzer. The two overlapped area is the linear polarization along the ∣*V*〉 direction, which will be blocked by the analyzer, while the nonoverlapped edge area is the circular polarization and will pass through the analyzer. Last, the corresponding edge images along the different phase gradient directions are shown clearly in Fig. 4 (I to L). As shown, the corresponding edge resolution is equal to around 100 μm. All images are acquired by the sum of 300 times accumulation with 2-s exposure duration of each frame, where the camera is triggered by photons of the heralding arm in different polarizations to record a normal or an edge image on the ICCD. Clearly, the switch demonstrated here is a nonlocally positioned switch, which is achieved by using the polarization correlation of quantum entanglement.

**Fig. 5:**
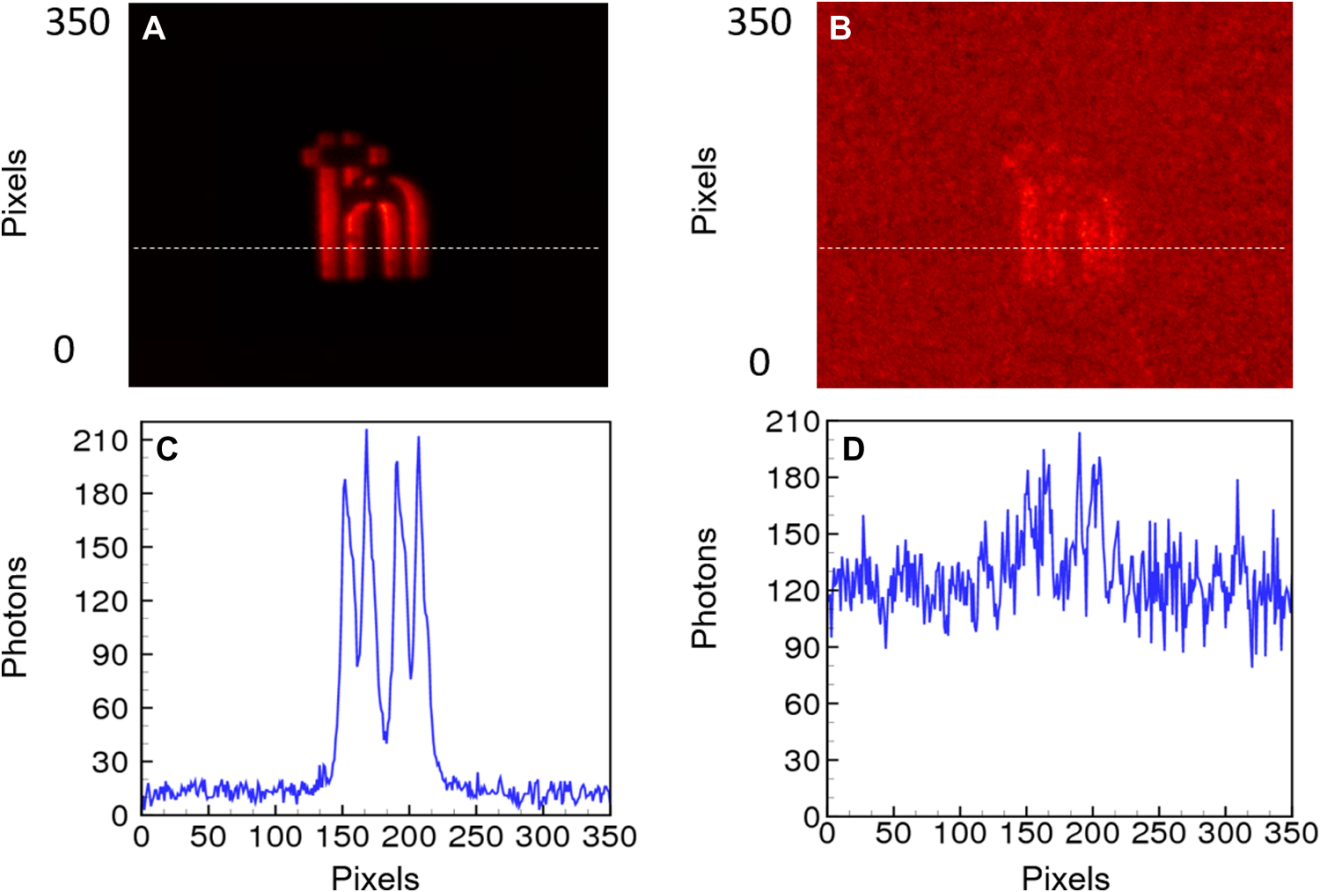
Entanglement-enabled quantum edge detection has high SNR. (**A** and **C**) The edge detection images are triggered by the heralding detector. (**B** and **D**) Direct images where the ICCD is internally triggered. (C) and (D) are taken along the white dashed lines in (A) and (B), respectively.

In addition to the aforementioned switchable capability, the quantum edge detection scheme owns another advantage of high SNR. By exploiting the strong temporal correlation of energy time–entangled photon pairs, the ambient noise can be notably reduced via second-order temporal coincidence measurements by two individual photodetectors (*49*). In our system, the noise is accumulated only within a very short time window when heralding signal triggers, which prevents almost all noise photons from falling into the effective coincidence (refer to note S2). While in classical optics, the noise will be continuously accumulated. In Fig. 5A, the edge image is acquired by the external trigger with remarkable SNR (refer to note S3). For comparison, direct imaging is measured, where the camera is exposed continuously. The effective exposure time of the direct imaging is set to the same value as the external trigger case. Besides, other equivalent experimental conditions are also used for both cases including CW source, low photon flux, and signal photon level comparable to background photon noise. The result is shown with a very low SNR in Fig. 5B, since both signal photons and background photons are detected indiscriminately in direct imaging configuration. Figure 5 (C and D) shows the cross section intensity distribution along the dashed lines in Fig. 5 (A and B), respectively. At least one order of magnitude SNR improvement is obtained in our entanglement-enabled quantum edge detection experiment. Alternatively, a similar SNR could also be achieved if a pulsed laser is used with the sync aligned to the camera, but this lacks nonlocality.

## DISCUSSION

It is known that one can also achieve edge detection by using a traditional amplitude filter placed in the Fourier plane of a 4f system. However, several advantages of our metasurface should be addressed here compared with traditional amplitude filters. First, the predesigned metasurface here is polarization dependent, which can be, fortunately, combined with the polarization-entangled source to show nonlocal switch performance. Notably, polarization-independent conventional filters such as simple amplitude filters are not able to activate the nonlocal switch. Instead of regular mode and edge mode achievement, they can only work at image edge mode when stationarily placed in the 4f imaging system. In addition, metasurfaces enable ultrathin and lightweight optical elements with precisely engineered phase profiles for obtaining various functions, wherein it is easier to replace traditional optical elements for a more compact and integrated system. Therefore, it is essential to integrate two metalenses and one metasurface spatial differentiator into a monolithic compound flat optic to achieve the same function, which also could not be obtained by traditional amplitude filters.

In this work, by using the polarization correlation of quantum entanglement, a nonlocally positioned switch for switchable edge detection is demonstrated. This conception may open new opportunities for security applications including image encryption and steganography, since a certain image mode (edge mode or regular mode) can only be distilled down from incident-mixed image modes by properly manipulating the remote switch with aligned external triggers for synchronization. In addition, the higher SNR enabled by nonlocal temporal correlations is of great benefit to photon-hungry scenarios such as tracking of enzymatic reactions and observing living organisms or photosensitive cells. With the exposure to intense light, thermal and electrical processes can affect their properties or even cause irremediable damage. Therefore, low-invasiveness optical probing will be a practical solution for ease of operation while maintaining time-tracking capacities. However, the impact of shot noise becomes more relevant in the low-light regime, compromising the SNR. Therefore, the scheme shown in this work will lay a foundation for the solution to this existing problem.

In conclusion, we have experimentally demonstrated that a polarization-entangled photon source can be used as a nonlocal switch for an optical edge detection mode without any alteration of the 4f imaging system based on a high-efficiency dielectric metasurface. By projecting idler photons in the heralding arm to ∣*H*〉 or ∣*V*〉, it will selectively trigger the ICCD to detect either a normal image or an edge image, respectively, which could be regarded as a quantum edge detection switch. This quantum edge detection scheme also provides a new suggestion for secure image communication such as encryption and steganography, which is impossible to achieve by using classical sources. From another point of view, our remotely switchable edge detection scheme using heralded single-photon imaging technique offers appealing SNR, although not beyond the shot noise limit, indicating advantages for various photon-hungry imaging and sensing applications.

## MATERIALS AND METHODS

### Information about ICCD

ICCD, Andor iStar A-DH334T-18 U-73; quantum efficiency, ~20%; effective pixel size, 13 μm × 13 μm; maximum of 500-kHz triggering rate; air cooling to −30°C. It should be noted that there are two important factors related to the ICCD operation, which are the intensifier gate width and the exposure time. The former is used to amplify the input photon by intensifier, of which the duration is typically several nanoseconds. The latter one, exposure time, is the length of time exposed to light between each readout of the CCD chip, typically a few seconds. Obviously, the signal or photon could be fired many times by an intensifier during each exposure time. Therefore, each readout frame can be regarded as a summary of all the detected single photons obtained during the exposed events. In this work, the intensifier of the ICCD camera is triggered by external pulses with a gate width of 4-ns duration. The ICCD system is operated with a constant gain of 3000 that corresponds to the intensifier voltage set as 0.6 V.

### Object mask fabrication

To demonstrate the quantum edge detection, we fabricate the object pattern through a markless photolithography process. The pattern information is designed and created by an auto computer-aided design software (Klayout), which is transformed into a Graphic Design System format to fix drawing errors created during the design. A 2.5-inch fused quartz gelatin chromium plate with 3-mm thickness is used as a lithography photomask for object fabrication. This plate is already precoated with a 100-nm-thick chrome layer and a 560-nm-thick positive resist film. First, ultraviolet exposure at a 405-nm working wavelength is performed on the photomask blank with an Advantools maskless photolithography (ATD1500). The resist film is then patterned in accordance with the predetermined pattern. Later on, development is carried out in a developer (AZ300MIF) for 50 s with agitation to wash away the photoresist in the exposed area. Then, the chrome is etched away where the resist is clear by an etching solution of Ce(NH_4_)_2_(NO_3_)_6_ for 90 s. The remaining photoresist is removed by piranha solution, and then the whole cleaning process is performed by using ultrapure water to swill the sample for 60 s before drying under an air gun. Last, the pattern transfer process is completed on the photomask.

## Supplementary Material

http://advances.sciencemag.org/cgi/content/full/6/51/eabc4385/DC1

Adobe PDF - abc4385_SM.pdf

Metasurface enabled quantum edge detection

## SUPPLEMENTARY MATERIALS

Supplementary material for this article is available at http://advances.sciencemag.org/cgi/content/full/6/51/eabc4385/DC1
