# Supplementary material for: Metasurface enabled quantum edge detection
Source: Sci Adv. 2020 Dec 16;6(51):eabc4385. doi: 10.1126/sciadv.abc4385 (PMC7744082; doi:10.1126/sciadv.abc4385)
Supplement: http://advances.sciencemag.org/cgi/content/full/6/51/eabc4385/DC1 [file supp_6_51_eabc4385__index.html]

Science Advances | Science AdvancesAAASSearchScience AdvancesMenu

## Supplementary Materials

# Metasurface enabled quantum edge detection

Junxiao Zhou, Shikai Liu, Haoliang Qian, Yinhai Li, Hailu Luo, Shuangchun Wen, Zhiyuan Zhou, Guangcan Guo, Baosen Shi, Zhaowei Liu

Download Supplement

**This PDF file includes:**

- Notes S1 to S3
- Figs. S1 to S3

**Files in this Data Supplement:**

- Adobe PDF - abc4385\_SM.pdf
