# Supplementary material for: Metasurface enabled quantum edge detection
Source: Sci Adv. 2020 Dec 16;6(51):eabc4385. doi: 10.1126/sciadv.abc4385 (PMC7744082; doi:10.1126/sciadv.abc4385)
Supplement: http://advances.sciencemag.org/cgi/content/full/6/51/eabc4385/DC1 [file supp_6_51_eabc4385__1.pdf]

Science Advances
AAAS

advances.sciencemag.org/cgi/content/full/6/51/eabc4385/DC1

Supplementary Materials for

Metasurface enabled quantum edge detection

Junxiao Zhou, Shikai Liu, Haoliang Qian, Yinhai Li, Hailu Luo*, Shuangchun Wen, Zhiyuan Zhou*, Guangcan Guo, Baosen Shi, Zhaowei Liu*

*Corresponding author. Email: zyzhouphy@ustc.edu.cn (Z.Z.); hailuluo@hnu.edu.cn (H.L.); zhaowei@ucsd.edu (Z.L.)

Published 16 December 2020, *Sci. Adv.* **6**, eabc4385 (2020)
DOI: 10.1126/sciadv.abc4385

This PDF file includes:

Notes S1 to S3
Figs. S1 to S3

Supplementary Text

Note S1. Simulation of a regular "solid cat" and an edge enhanced "outlined cat".

A basic principle of a metasurface enabled "solid cat" and an edge enhanced "outlined cat", as shown in Fig. S1. A transmissive input image, "Schrödinger's cat" as an object, is illuminated by a plane wave. As shown in Fig. S1, the first lens (L_1) computes the Fourier transform of the electric field at the object plane. The metasurface is located at the Fourier plane of the 4f system, where the Fourier spectrum is formed. The second lens (L_2) takes the inverse Fourier transform of the spectrum modified by the metasurface and creates the output edge information at its back focal plane. To illustrate the calculation process, assume the incident light is x-polarized, the input electric field of the object is defined as $E_0(x_1, y_1)\begin{pmatrix}1\\0\end{pmatrix}$. After propagating through L_1, the electric field $E_1(r_1,\theta_1)$ right in front of the metasurface is given as:$E_1(x_2, y_2) = \mathcal{F}[E_0(x_1, y_1)]$, where $\mathcal{F}$ is the Fourier transform operator. Considering the spatial differentiation function of the metasurface, the electric field right behind the metasurface is $E_2(x_2, y_2) = E_1(x_2, y_2)\ \left[\exp\left(i * \frac{2\pi}{\Lambda} * x\right)\begin{pmatrix}1\\-i\end{pmatrix} + \exp\left(-i * \frac{2\pi}{\Lambda} * x\right)\begin{pmatrix}1\\i\end{pmatrix}\right]$. Here, Λ is the period of the metasurface; these two terms, $\exp\left(i * \frac{2\pi}{\Lambda} * x\right)$ and $\exp\left(-i * \frac{2\pi}{\Lambda} * x\right)$ are the PB phase achieved by metasurface for LCP and RCP components; for terms, $\begin{pmatrix}1\\i\end{pmatrix}$ and $\begin{pmatrix}1\\-i\end{pmatrix}$, these are Jones vectors for LCP and RCP components.

To achieve the edge information, two orthogonal polarizers are employed in our system. Therefore, the electric field $E_2(x_2, y_2)$ could be further given as $E_2(x_2, y_2) = E_1(x_2, y_2)\ \left[\exp\left(i * \frac{2\pi}{\Lambda} * x\right) - \exp\left(-i * \frac{2\pi}{\Lambda} * x\right)\right] = E_1(x_2, y_2)\sin\left(\frac{2\pi}{\Lambda} * x\right)$. After the propagation through L_2, the electric field at the image plane is derived from $E_3(x_3, y_3) = \mathcal{F}[E_2(x_2, y_2)]$. The light intensity of the output image is given as $I_{out} = \left|E_3(x_2, y_2)\right|^2$. As shown in Fig. S2C, the edges end up with an "outlined cat".

It should be noted that for our imaging system, if two orthogonal polarizers are changed to co-polarized state or the second polarizer is removed, the mentioned electric field $E_2(x_2,y_2)$ behind the metasurface will be modified as $E_2(x_2,y_2) = E_1(x_2,y_2)\ \left[\exp\left(i * \frac{2\pi}{\Lambda} * x\right) + \exp\left(-i * \frac{2\pi}{\Lambda} * x\right)\right] = E_1(x_2,x_2)\cos\left(\frac{2\pi}{\Lambda} * x\right)$. After propagation through L_2, the electric field at the image plane is written as $E_3(x_3,y_3) = \mathcal{F}[E_2(x_2,y_2)]$. The final intensity distribution is given as $I_{out} =$

$\left|E_3\left(x_{2,}y_2\right)\right|^2$. We can expect that a complete "solid cat" with the half-intensity of the edges will be obtained as demonstrated in Fig. S2D.

The above discussion all focuses on the classical circumstance. If we replace the linearly polarized illumination source with unknown states of photons from polarization entanglement and remove the P1, the image will be a superposition state, a "Schrödinger's cat" state consisting of both "outlined cat" and "solid cat", which will be discussed specifically in the introduction of the manuscript.

Note S2. The measurement details

Fig. 5 shows a clear comparison between heralding image and direct image. As for quantum heralded imaging in Fig. 5A and Fig. 4, the ICCD operates at an **external trigger** mode and a **Digital Delay Generator (DDG)** gate mode where the photocathode is switched on only when the gate pulse from the DDG (trigger signal) is high, which allows the coincidence measurement of time-correlated photon pairs. The measurement conditions in this mode are 2s discrete exposure of each frame, 300 accumulative times (frame number), 4ns gate width. Due to the very high simultaneity of twin photon pairs generated through SPDC, the use of heralded single photons ensures that the background counts can be virtually eliminated from the recorded images in a very narrow coincidence time-window, leading to a very high SNR.

As for classical direct image in Fig. 5B, the ICCD operates at an **internal trigger** mode and a **Fire Only** gate mode where the photocathode is switched on during the whole exposure time. The gate width (fire pulse length) equals to the total continuous exposure time that is 0.11s for single frame capture (see calculation below). At this work mode, both signal photons and background photons can be detected indiscriminately during the whole acquisition time, which causes the SNR decrease dramatically. To ensure the conditions of wavelength, bandwidth, and photon flux are exactly equal in both cases, the illumination source is polarized and purified photons from the imaging path of the entangled photon source.

In both cases, equivalent experimental conditions are used. For demonstrating the anti-noise capacity of quantum edge detection, the ambient light keeps at roughly the same light level as the signal, which means the noise and signal could at the same order of magnitude. Experimentally, we kept the laboratory in dark except the ambient light scattered by a display screen. The total illumination photon counts before the object are roughly 2.4×10^5/s and the total average environmental noise counts detected per pixel is roughly 1.1×10^3 Hz when operates at continuous exposure. It should be noted that the noise source is primarily from the background, not from dark counts of ICCD in our experiment. Guided by the official specification of the camera, dark signal, a charge usually expressed as a number of electrons N, is produced by the flow of dark current. According to the specification sheet, the dark current per second per pixel under the 30° C air cooling condition is 0.1Hz electron. The shot noise is the square root of N, which is 0.32 Hz electron, roughly equivalent to 6.3×10^{-4} Hz photon, which is several orders of magnitude smaller

than the ambient light. The calculating equation of the total effective exposure time T_{eff} is given below:

$$T_{eff} = CC * t_s * t_g * N$$

Where CC is the coincidence counts per second, which is roughly 4.4×10^4/s. t_s is 2s exposure time of each frame. t_g is 4 ns gate width and N is the number of frames, which is 300 times. Finally, we got an effective exposure time where $T_{eff} \approx 0.11$ s in both cases. At the final stage, the acquired raw data from ICCD is extracted pixel by pixel using the software of Mathematica and then all the performed data is imported to the software of Tecplot for color rendering.

Note S3. Noise analysis

1. Theory

To analyze the signal-to-noise ratio (SNR) of image performance, we now have a brief theoretical analysis and calculation of the quantum heralded source characteristic. For the sake of simplicity, three reasonable hypotheses are put forward. First, optical scattering during the imaging process is negligible. Second, the ICCD camera is regarded as a single-pixel detector. Third, the probability of multi-photon (>2) emission events is neglected due to stimulated emission via low SPDC pump power. We start by discussing the SNR of our proposed quantum edge detection scheme. The coincidence probability of correlated photons R_{cc} is given by $R_{cc} = \mu_c \alpha_s \alpha_i$, where μ_c is the photon-pair generated probability and $\alpha_{s,i}$ denotes the total efficiency in the signal and idler arm, respectively, including the quantum efficiencies of the instruments, the transmission efficiency, as well as the collection efficiency of fibers. The count probabilities of a single event at the signal path detected by ICCD or the idler path detected by SPAD are given below:

$$N_s = (\mu_c + \mu_s)\alpha_s + d_s$$

$$N_i = (\mu_c + \mu_i)\alpha_i + d_i$$

where $\mu_{s,i}$ denotes the probability of environmental noise in signal-channel and idler-channel, respectively. $d_{s,i}$ is the dark count probability caused by ICCD and SPAD, respectively.

The effective signals (S_Q) of the image in proportion to the probability of coincidences (R_{cc}) can be expressed as $S_Q \propto R_{cc} = \mu_c \alpha_s \alpha_i$. Likewise, the unwanted background light and sensor noise (N_Q) in proportion to the probability of accidental events (A) can be expressed as $N_Q \propto A = N_s N_i$. Finally, the SNR in a coincidence image using heralded single photon imaging modality is derived below:

$$SNR_Q = \frac{S_Q}{N_Q} = \frac{\mu_c \alpha_s \alpha_i}{[(\mu_c + \mu_s)\alpha_s + d_s][(\mu_c + \mu_i)\alpha_i + d_i]} \quad \text{(S1)}$$

Then we characterize the SNR of conventional classical images with direct measurements. Herein, first order intensity measurement is carried out by a single detector (i.e. camera sensor). The effective signal intensity is given by $S_D \propto \mu_c \alpha_s$, and the total noise including background light and sensor noise is given by $N_D \propto \mu_s \alpha_s + d_s$. Therefore, the image SNR using direct imaging modality can be written as

$$SNR_D = \frac{S_D}{N_D} = \frac{\mu_c \alpha_s}{\mu_s \alpha_s + d_s} \quad \text{(S2)}$$

From equations (S1) and (S2), we can easily see the distinct working mechanisms of these two types of imaging modalities. For quantum heralded imaging, one effective photon event can only be obtained by second order intensity joint detection using two independent detectors (i.e. SPAD and camera sensor). Besides, the probability of a coincidence noise event is also significantly reduced via joint-detections, which could benefit from the preferential rejection of individual noise events. For direct imaging, first order intensity measurement is performed by using only a single detector (i.e. camera sensor). This modality makes no distinct detection between signals and noise, finally causing a pretty lower SNR. For a better understanding, the corresponding simulations are also given in the following section.

2. Calculation

By using the derived equation as the analytical model, we have a rough simulation of the SNR function in both heralded coincidence and conventional cases, shown in Figs. S3 (A to C) and Figs. S3 (D to F) respectively. From Figs. S3 (A and D), in the condition of low light level illumination, we can see that quantum heralded imaging via coincidence measurements promises advantages over direct imaging in terms of SNR. However, quantum superiority can be canceled by significantly increasing the power of the illuminated source, which means the single-photon property of the light source is completely degraded. From Figs. S3 (B and E), the variation trend of SNR as a function of α_s is monotonic increasing in both cases. That means if one has a better instrument (ICCD or SPAD) with higher quantum efficiency, the SNR will improve to a certain extent. As shown in Figs. S3 (C and F), the image SNR can be degraded by the increase of background noise. Even though, the quantum heralded SNR can still beat its conventional counterpart, suggesting promises in low light level detection. It should be noted that the SNR achieved in our experiment cannot reach the theoretical simulation limited by several main factors including environmental disturbance, optical scattering, optical device imperfections, and so on.

Fig. S1. The photos of illuminated objects

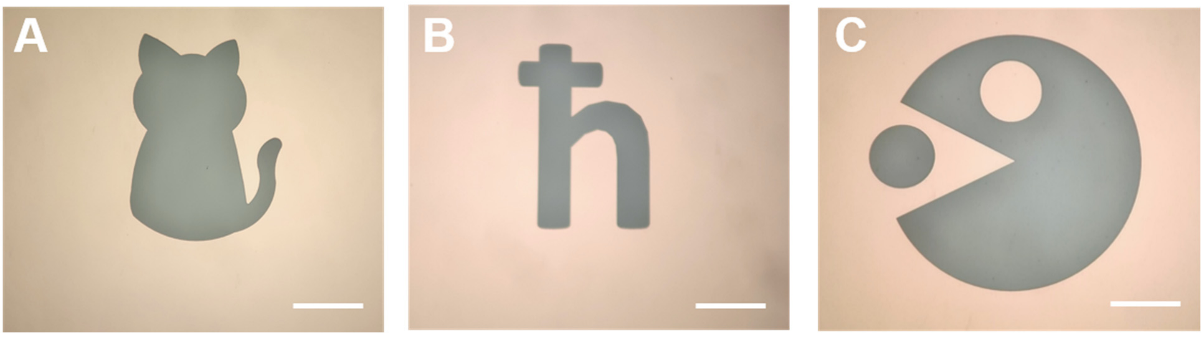

Fig. S1 The objects employed for this work. Scale bar, 500 μm.

Fig.S2. The simulation result of metasurface enabled quantum edge detection

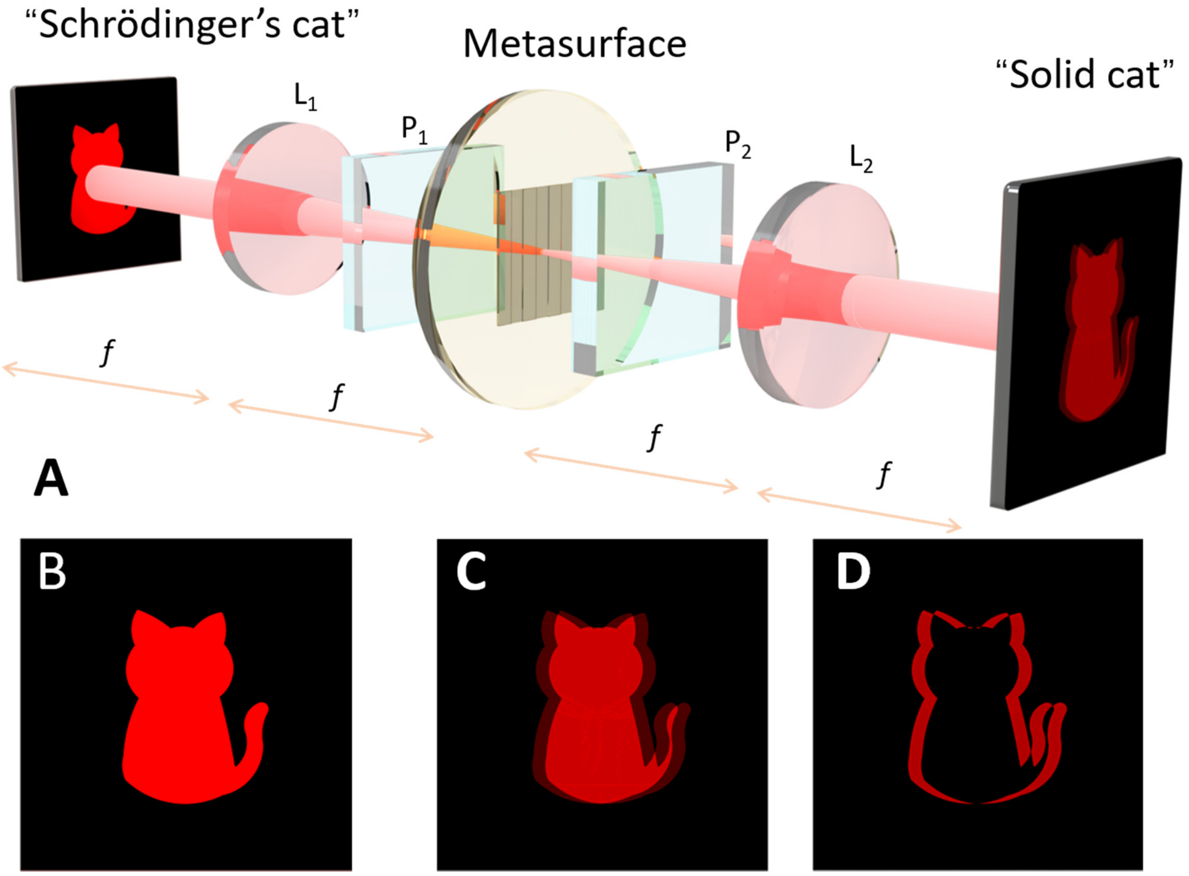

Fig. S2 The simulation result of metasurface enabled quantum edge detection. (**A**) Partial experiment setup of the imaging arm. (**B**) The "Schrödinger's cat". (**C**) A regular mode of a "solid cat". (**D**) An edge detection mode of an "outlined cat".

Fig. S3 Calculation results of SNR function.

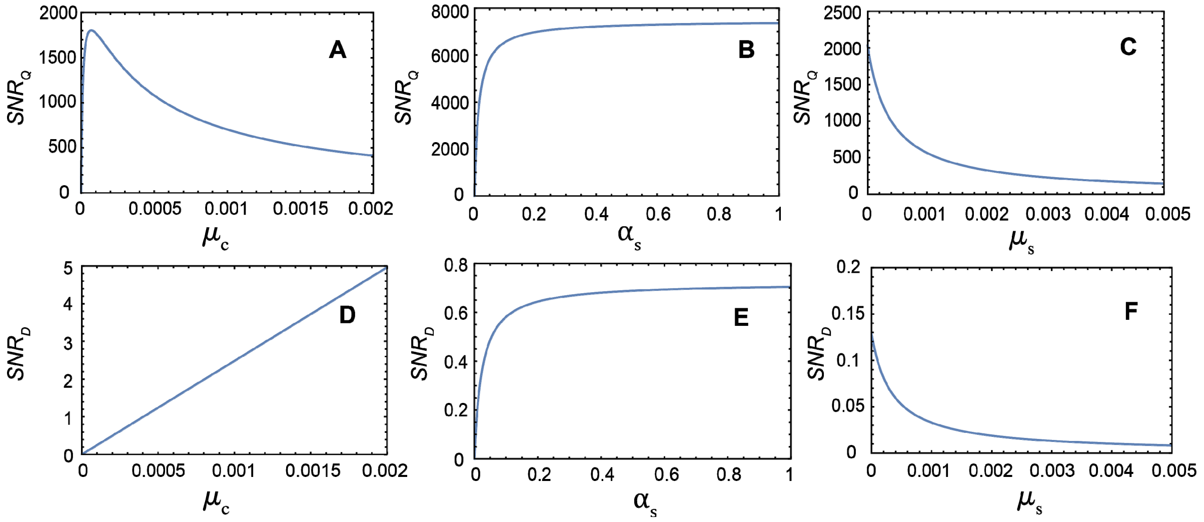

Fig. S3. Calculation results of SNR function. (**A** and **D**) show the SNR as a function of μ_c, the probability of photon pairs. (**B** and **E**) show the SNR as a function of α_s, the total efficiency of the signal path. (**C** and **F**) show the SNR as a function of μ_s, the probability of background noise in the signal path. (**A** to **C**) The simulated SNR function in the quantum heralded case. (**D** to **F**) The simulated SNR function in the case of conventional direct measurement. The experimental parameters used for the calculation are given below: $\alpha_s = 0.43\%$; $\alpha_i = 15\%$; $\mu_s = 6.1 * 10^{-5}$; $d_s = 1.47 * 10^{-6}$; $\mu_i = 2.33 * 10^{-6}$; $d_i = 1.47 * 10^{-6}$; $\mu_c = 4.4 * 10^{-5}$.
